# Persistent abnormalities in pulmonary arterial compliance after heart transplantation in patients with combined post-capillary and pre-capillary pulmonary hypertension

**DOI:** 10.1371/journal.pone.0188383

**Published:** 2017-11-27

**Authors:** Stefano Ghio, Gabriele Crimi, Silvia Pica, Pier Luigi Temporelli, Massimo Boffini, Mauro Rinaldi, Claudia Raineri, Laura Scelsi, Massimo Pistono, Rossana Totaro, Stefania Guida, Luigi Oltrona Visconti

**Affiliations:** 1 Division of Cardiology, Fondazione IRCCS Policlinico S. Matteo, Pavia, Italy; 2 Division of Cardiology, Istituti Clinici Scientifici Maugeri, Veruno, Italy; 3 Division of Cardiac Surgery, Surgical Sciences Department, Città della Salute e della Scienza, University of Turin, Turin, Italy; Scuola Superiore Sant'Anna, ITALY

## Abstract

**Background:**

The hemodynamic definitions of pulmonary hypertension (PH) in left heart disease have recently been refined to better match the characteristics required to reflect the presence of pulmonary vascular disease. Accordingly, we tested the hypothesis that abnormalities in the stiffness of pulmonary circulation would persist after heart transplantation in patients with combined post-capillary and pre-capillary PH (Cpc-PH) in contrast to those with isolated post-capillary PH (Ipc-PH).

**Methods:**

We retrospectively analyzed right heart hemodynamics in a cohort of 295 consecutive patients with heart failure and advanced left ventricular systolic dysfunction (LVSD) before and 1 year after heart transplantation.

**Results:**

According to their baseline hemodynamic profile, patients were classified as: 75 Cpc-PH, 111 Ipc-PH, and 98 without PH (no-PH), and 11 pre-capillary PH. One year after heart transplantation, pulmonary artery pressures, pulmonary vascular resistance and cardiac index normalized in all patients regardless of the baseline hemodynamic profile. However, pulmonary arterial compliance remained lower in Cpc-PH patients (from 1.6±1.2 at baseline to 3.7±1.4 ml/mmHg at 1 year) than in Ipc-PH (from 1.2±2.0 to 4.4±2.3 ml/mmHg) and no-PH patients (from 3.7±2.0 to 4.5±1.8 ml/mmHg); (adjusted p = 0.03 Ipc-PH vs. Cpc-PH _INT_<0.001).

**Conclusions:**

In heart failure patients with advanced LVSD, a hemodynamic profile characterized by Cpc-PH predicts the persistence of a stiffer pulmonary circulation at 1 year after heart transplantation.

## Introduction

The hemodynamic definitions of pulmonary hypertension (PH) in left heart disease have recently been modified in the European Guidelines to better match the characteristics required to reflect the presence of pulmonary vascular disease. Definitions now include the diastolic pressure gradient (DPG), reported to be a more stable hemodynamic parameter than pulmonary vascular resistances (PVR) or transpulmonary gradient (TPG) [1]. PVR and TPG alone may in fact over-diagnose or under-diagnose pulmonary vascular disease in left heart diseases associated with increased pulmonary venous pressure, since these metrics vary with cardiac output and left atrial pressure [2,3].

Pulmonary arterial compliance (PCa) is increasingly recognized as a parameter of clinical and prognostic relevance in patients with different classes and forms of PH. A reduced PCa is a determinant of poor functional capacity in chronic thromboembolic PH [4,5] and it is a marker of poor prognosis in patients with pulmonary arterial hypertension [6–9]. It has been suggested that a reduced PCa may be the most important hemodynamic feature in early stages of pre-capillary forms of pulmonary hypertension [10]. And it has been shown that a reduced PCa is of prognostic value in heart failure patients, both in those with elevated PVR and in those with normal PVR [11–14].

We tested the hypothesis that in heart failure patients with a hemodynamic profile characterized by combined post-capillary and pre-capillary PH (Cpc-PH), abnormalities in the stiffness of the pulmonary circulation would persist at 1 year after heart transplantation despite normalization of pulmonary artery wedge pressure. PCa was considered as the primary candidate parameter to reflect potential persistent hemodynamic alterations given the evidence of its critical prognostic role in all forms and stages of PH. The study took advantage of the unique opportunity offered by heart transplantation to observe the effects of a long lasting normalization of left ventricular filling pressures in patients with advanced heart failure.

## Methods

### Study design

We performed a retrospective analysis of right heart hemodynamics in a cohort of 295 patients with advanced left ventricular systolic dysfunction (LVSD) who underwent heart transplantation and, before and 1 year after surgery, right heart catheterization.

### Patients

Consecutive patients who survived at least one year after heart transplantation were included.

The inclusion criteria were left ventricular ejection fraction ≤ 35% and etiology due to ischemic or hypertensive heart disease or idiopathic dilated cardiomyopathy. Exclusion criteria were: organic valvular heart disease, previous surgery for valvular heart disease, other cardiomyopathies (such as restrictive or hypertrophic cardiomyopathy and arrhythmogenic right ventricular cardiomyopathy).

Before transplantation, patients underwent right heart catheterization as part of the diagnostic protocol for heart failure evaluation and transplantation eligibility assessment; after transplantation, patients underwent the hemodynamic evaluation as part of the diagnostic protocol for the monitoring of organ rejection (which includes endomyocardial biopsy). None of the transplant donors were from a vulnerable population and all donors or next of kin provided written informed consent that was freely given. The investigation complied with the principles outlined in the Declaration of Helsinki; patients signed an informed consent and the study was approved by the Institutional Review Board of the Fondazione IRCCS Policlinico S.Matteo for observational, non-pharmacological, non-sponsored studies which complies with the Italian legislation on privacy (Codex on the Privacy, D. Lgs. 30/06/2003, n. 196).

The following right heart catheterization data were collected: pulmonary artery wedge pressure (PAWP); systolic, diastolic and mean pulmonary artery pressure (mPAP); pulmonary pulse pressure (PP); cardiac output (CO) evaluated by thermodilution; cardiac index (CI); stroke volume (SV) obtained dividing cardiac output by heart rate); right atrial pressure (RAP); TPG, calculated as mPAP—PAWP; and DPG calculated as diastolic PAP—PAWP; PVR, calculated as TPG/CO. PCa was estimated dividing the blood volume driven from each heart beat in the pulmonary vascular tree, namely the SV, by the corresponding change in the pulmonary artery pressure: PCa ≈ SV/PP [ml/mmHg]. According to the 2016 European Guidelines, Cpc-PH was defined as mPAP≥25 mmHg, PAWP>15 mmHg, DPG≥7 mmHg and/or PVR>3 WU, and isolated post-capillary PH (Ipc-PH) as mPAP≥25 mmHg, PAWP>15 mmHg, DPG<7 mmHg and/or PVR≤3 WU.

### Statistical analysis

Continuous data are presented as mean ± SD. Absolute changes in study endpoints with regard to 1 year post transplant were analyzed using an ANOVA with repeated measures and the Tukey honest significant difference (HSD) test was used to adjust for multiple comparisons. An improvement in pulmonary vascular compliance was defined as a compliance at follow-up (1 year) which is greater than at baseline. Statistical significance was set at a level of p < 0.05. All statistical analyses were performed using R software (http://www.R-project.org/, version 3.1.0).

## Results

### Hemodynamic changes after transplantation in the whole cohort

**Fig 1:**
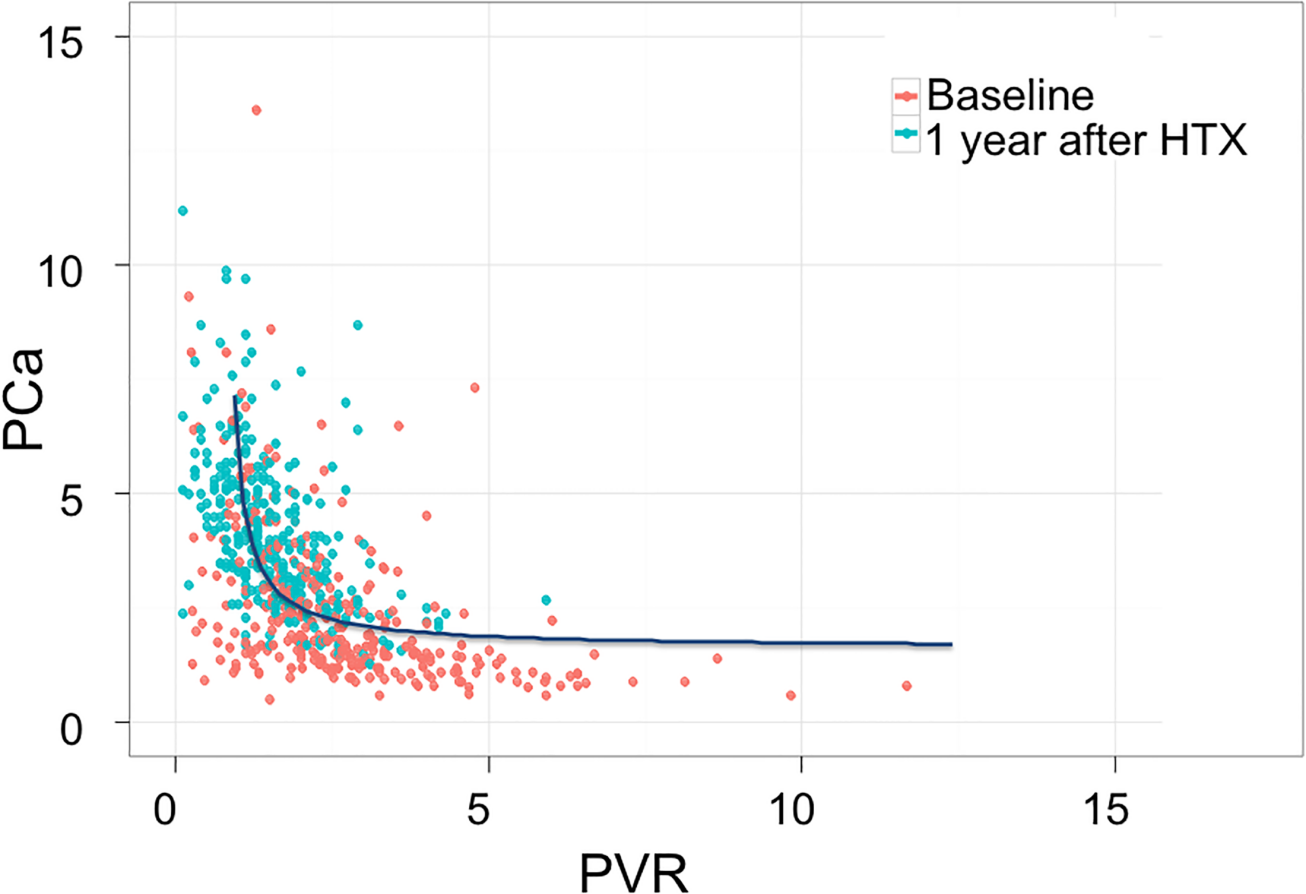
PCa vs. PVR data at baseline and after transplant in the entire cohort. Red dots indicate baseline values, green dots data at 1 month and blue dots data at 1 year after transplant.

Overall, the baseline right heart hemodynamic profile was characterized by low CI, mild PH and slightly elevated PVR, with only a few (n = 14) patients who had a high DPG (i.e. ≥7 mmHg). Table 1 shows patients’ hemodynamic data at baseline and at 1 year and the corresponding delta. In brief, CI normalized at 1 year after heart transplantation as well as systolic, diastolic and mean PAP and PVR; PCa increased significantly. Systemic blood pressure also substantially increased but systemic compliance did not change. The relationship between PCa and PVR showed a shift upward and leftward at 1 year after surgery (Fig 1).

**Table 1:** Hemodynamic parameters in the whole cohort.

	Baseline	1 year	Δ *1 year—baseline*	time effect
	*n = 295*	*n = 295*		p-value
Systolic BP, mmHg	103.8 (15.9)	137.1 (15.6)	33.4 (21.3)	<0.001
Diastolic BP, mmHg	67.5 (11.7)	88.4 (12.1)	21.1 (16)	<0.001
Heart Rate, bpm	76.3 (14.7)	84.0 (11.4)	7.9 (17.2)	<0.001
Mean BP, mmHg	79.6 (11.9)	104.3 (13.5)	24.8 (17.1)	<0.001
Systolic PAP, mmHg	43.2 (14.7)	24.5 (6.4)	-18.7 (14.7)	<0.001
Mean PAP, mmHg	29.6 (10.2)	15.1 (4.8)	-14.5 (10.6)	<0.001
Diastolic PAP, mmHg	20.4 (8.5)	7.9 (4.5)	-12.5 (9.2)	<0.001
TPG, mmHg	8.4 (5.1)	7.7 (3.7)	-0.7 (5.9)	0.075
DPG, mmHg	-0.8 (5)	0.5 (3.5)	1.3 (6.2)	<0.001
Cardiac Index, L/min/m^2^	1.9 (0.5)	2.9 (0.6)	1 (0.8)	<0.001
Cardiac Output, L/min	3.3 (0.9)	5.3 (1.2)	1.9 (1.4)	<0.001
RAP, mmHg	7.8 (6.2)	3.7 (3.4)	-4 (6.7)	<0.001
PAWP, mmHg	21.2 (9)	7.4 (4)	-13.8 (9.7)	<0.001
PVR, mmHg/(L/min)	2.8 (2.2)	1.6 (0.9)	-1.2 (2.3)	<0.001
Pulmonary Ca, ml/mmHg	2.5 (1.8)	4.3 (2.1)	1.7 (2.4)	<0.001
Systemic Ca, ml/mmHg	1.4 (0.6)	1.4 (0.5)	0 (0.7)	0.702
Stroke Volumeml	45.3 (15.5)	63.2 (14.8)	17.6 (18.6)	<0.001

BP = blood pressure; PAP = pulmonary artery pressure; TPG = transpulmonary gradient: DPG = diastolic pulmonary gradient; RAP = right atrial pressure; PAWP = pulmonary artery wedge pressure; PVR = pulmonary vascular resistance; Ca = compliance.

### Hemodynamic changes after transplantation in Cpc-PH vs. Ipc-PH

Seventy-five patients (25.4%) met the definition of Cpc-PH (mPAP>25 mmHg, PAWP>15 mmHg, DPG≥7 mmHg and/or PVR>3 WU), and 111 patients (37.6%) met the definition of Ipc-PH (mPAP>25 mmHg, PAWP>15 mmHg, DPG<7 mmHg and/or PVR≤3 WU). Ninety-eight patients (33.2%) had no PH. Eleven patients (3.7%) had a hemodynamic profile characterized by pre-capillary PH (mPAP>25 mmHg, PAWP≤15 mmHg).

**Fig 2:**
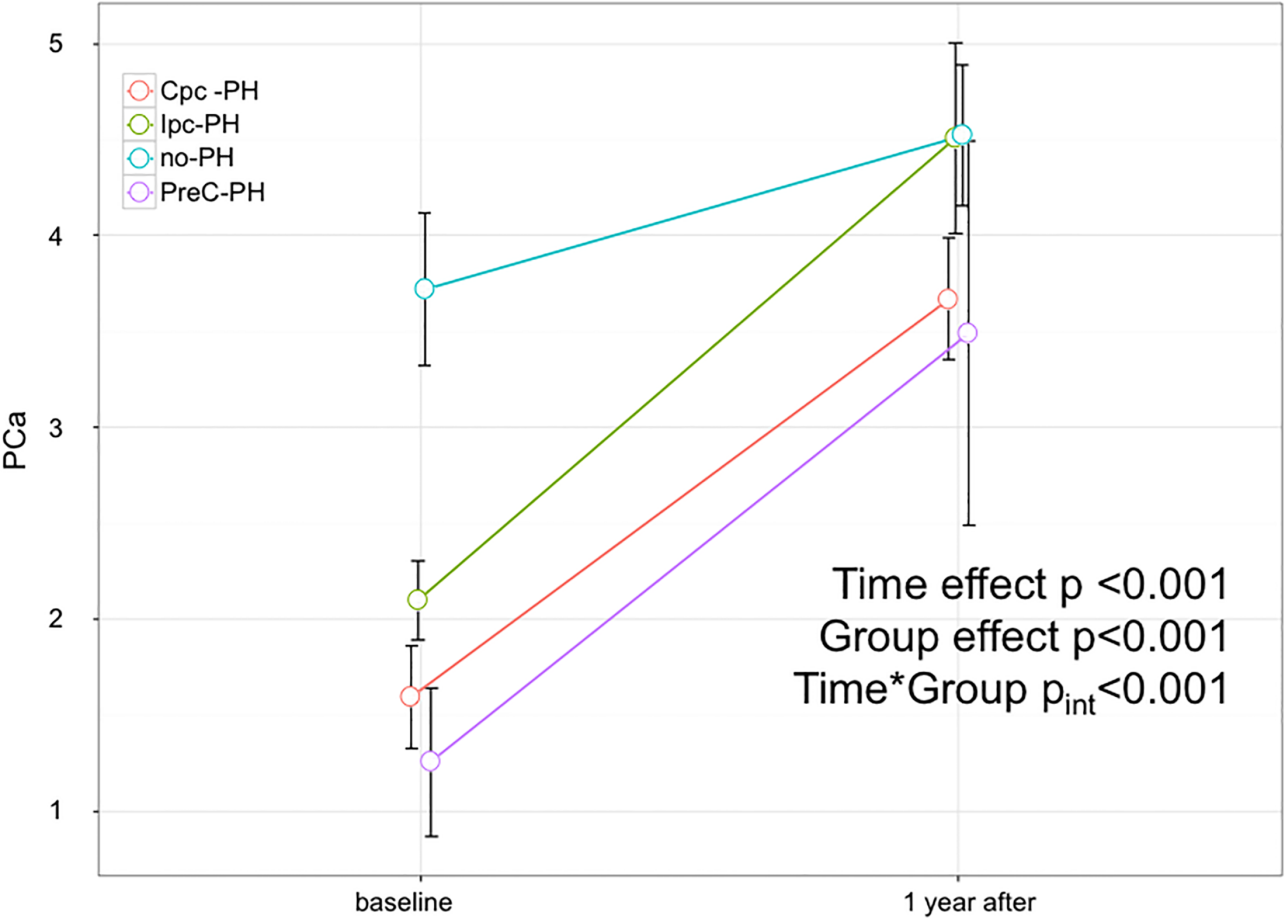
PCa changes in the different patient subgroups. Four hemodynamic subgroups are shown: Cpc-PH, Ipc-PH, patients without PH (no PH) and patients with pre-capillary PH (preC-PH).

**Fig 3:**
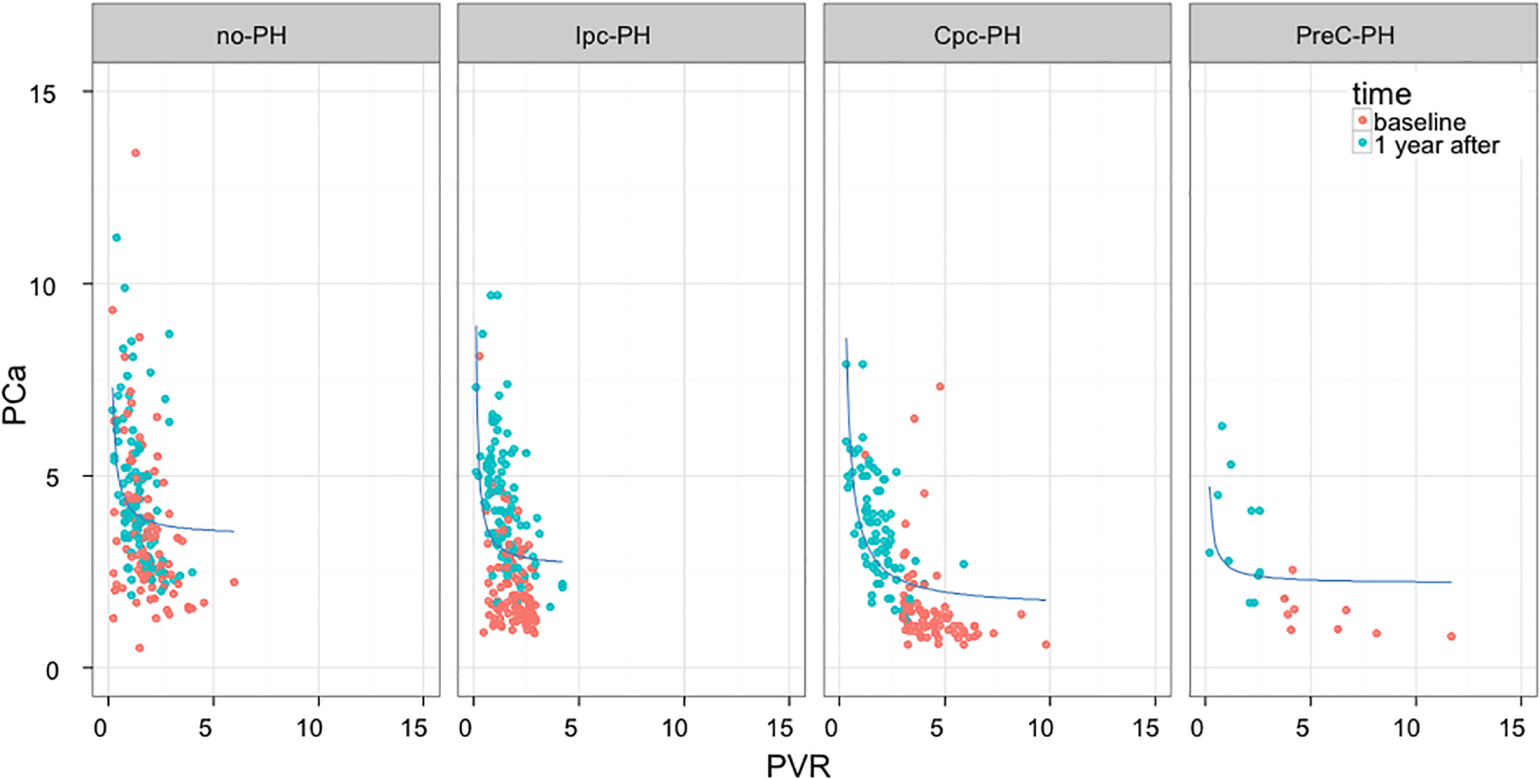
PCa vs. PVR data at baseline and 1 year after transplantation in the different subgroups. Four hemodynamic subgroups are shown: Cpc-PH, Ipc-PH, patients without PH (no PH) and patients with pre-capillary PH (preC-PH).

The hemodynamic data before and after transplantation in different hemodynamic subgroups are shown in Table 2. Regardless of the hemodynamic profile at baseline, CI normalized after heart transplantation as well as PAWP, mean PAP and PVR. Systemic compliance did not change but PCa improved (as shown in Fig 2). Fig 3 shows the scatterplot of individual PCa-PVR data at baseline and 1 year after transplantation in all subgroups of patients: after surgery, individual data were shifted upward and leftward along the regression line in the Cpc-PH and in the pre-capillary PH subgroups whereas there was an upward shift in the no-PH and the Ipc-PH subgroups.

**Table 2:** Hemodynamic parameters at baseline and at 1 year in different hemodynamic subgroups.

	Baseline				1 year				Time effect	Group effect
	No-PH	CpC-PH	Ipc-PH	PreC-PH	No-PH	CpC-PH	Ipc-PH	PreC-PH	P value	P value
	n = 98	N = 75	N = 111	N = 11	n = 98	N = 75	N = 111	N = 11		
Systolic BP, mmHg	106.8 (17.3)	103.7 (14.4)	100.9 (15.3)	109.3 (16)	137.3 (15.7)	138.5 (17.1)	136.5 (14.7)	135 (14.5)	0.18	<0.001
Diastolic BP, mmHg	68.8 (11.8)	67.5 (11.5)	66.5 (11.6)	69.5 (11.7)	88.5 (12.8)	89.9 (12.2)	87.3 (11.2)	93 (11.8)	0.24	<0.001
Heart Rate, bpm	71.4 (13.4)	79.6 (15.3)	77.7 (14.5)	81.7 (14)	82.4 (11.3)	85.2 (11.9)	84.4 (11.4)	85.2 (10.2)	<0.001	<0.001
Mean BP, mmHg	81.5 (12.7)	79.5 (11.4)	78 (11.1)	82.8 (12.4)	104.7 (12.9)	106.1 (12.6)	103.7 (11.1)	97.3 (34.1)	0.23	<0.001
Systolic PAP, mmHg	28 (7.9)	56.2 (10.9)	47.4 (8.1)	49.7 (16.2)	23.7 (5.4)	26.3 (7.1)	24 (6.6)	24.8 (6.1)	<0.001	<0.001
Mean PAP, mmHg	18 (5)	39.1 (6.3)	33.4 (4.8)	32.7 (8.8)	14.7 (4.4)	16.4 (5.2)	14.6 (4.9)	14 (3)	<0.001	<0.001
Diastolic PAP, mmHg	11.7 (4.7)	27.4 (6.5)	23.6 (5.3)	19.4 (7.6)	7.8 (4.6)	8.4 (4.3)	7.6 (4.6)	7.1 (3.4)	<0.001	<0.001
TPG, mmHg	6.3 (2.9)	12.2 (3.8)	6.3 (2.5)	20.6 (11.6)	7.2 (3.3)	8.7 (3.9)	7.3 (3.5)	7.2 (3.9)	<0.001	<0.01
DPG, mmHg	0 (3.2)	0.5 (5.3)	-3.5 (3.8)	7.3 (10.5)	0.3 (3.7)	0.8 (3.4)	0.4 (3.2)	0.3 (4.2)	<0.001	<0.001
Cardiac Index, L/min/m^2^	2.1 (0.5)	1.6 (0.4)	1.9 (0.4)	1.6 (0.3)	2.9 (0.6)	2.8 (0.6)	3 (0.6)	2.5 (0.7)	<0.001	<0.001
Cardiac Output, L/min	3.7 (1)	2.9 (0.8)	3.4 (0.9)	2.7 (0.7)	5.4 (1.2)	5.1 (1.1)	5.4 (1.3)	4.7 (1.4)	<0.001	<0.001
RAP, mmHg	4.7 (4.6)	9.3 (5.5)	9.4 (6.3)	10.3 (10.5)	3.9 (3.4)	3.6 (3.3)	3.8 (3.7)	2.3 (0.9)	<0.001	<0.001
PAWP, mmHg	11.7 (5.2)	26.8 (5.5)	27.1 (5.2)	12.1 (3.8)	7.5 (3.7)	7.6 (4.1)	7.3 (4.4)	6.8 (3.1)	<0.001	<0.001
PVR, mmHg/(L/min)	1.8 (1)	4.3 (1.4)	1.9 (0.7)	8.5 (6.6)	1.4 (0.7)	1.8 (0.9)	1.4 (0.8)	1.7 (0.9)	<0.001	<0.001
Pulmonary Ca, ml/mmHg	3.7 (2)	1.6 (1.2)	2.1 (1.1)	1.3 (0.6)	4.5 (1.8)	3.7 (1.4)	4.5 (2.6)	3.5 (1.5)	<0.001	<0.001
Systemic Ca, ml/mmHg	1.5 (0.5)	1.1 (0.5)	1.5 (0.7)	1 (0.5)	1.4 (0.5)	1.3 (0.5)	1.4 (0.4)	1.4 (0.5)	<0.001	0.98
Stroke Volume, ml	52.6 (15.9)	38.2 (12.6)	45 (14.4)	34.7 (12.9)	65.4 (14.5)	60.5 (15.5)	63.9 (14.2)	55 (16.9)	<0.01	<0.001

BP = blood pressure; PAP = pulmonary artery pressure; TPG = transpulmonary gradient: DPG = diastolic pulmonary gradient; RAP = right atrial pressure; PAWP = pulmonary artery wedge pressure; PVR = pulmonary vascular resistance; Ca = compliance.

## Discussion

The key finding of the present study is that 1 year after cardiac transplantation, PCa remained lower in Cpc-PH patients than in Ipc-PH patients and patients without PH, despite complete normalization of all hemodynamic parameters.

The measurement of pulmonary arterial compliance has recently gained interest, in particular in patients with PH, given its demonstrated clinical and prognostic significance. According to a simple but reliable model, the hemodynamics of the systemic and pulmonary arterial circulation may be described in terms of resistance (strongly dependent on vessel diameter and mainly located in the small arteries and arterioles) and compliance (mainly located in central capacitance arteries) [14,15]. The major difference between the systemic and the pulmonary circulation is that in the former almost all compliance is central, whereas in the latter the compliance is more equally distributed over the whole system. As long as pulmonary venous pressure is low, the fact that compliance is equally distributed over the whole circulatory system determines a fixed coupling between resistance and compliance [16]. This may explain the observation that in such patients treatment with disease-specific drugs does not change significantly the product of PVR and PCa [10]. In patients with heart failure, the relationship between PCa and PVR is more complex than in patients with pre-capillary PH. When elevated, the PAWP amplifies peripheral pulse reflection, thereby augmenting systolic and mean pulmonary pressures and leading to a decline in total PCa [17]. Importantly, although the changes in PCa have been studied in patients treated for acute decompensated heart failure as well as in patients with chronic heart failure undergoing assist device implantation [12,18], it has not yet been explored how PCa is affected by the long lasting normalization of right heart hemodynamics determined by successful cardiac transplantation.

The model of heart transplantation was already used 20 years ago to assess the nature of PH in patients with advanced congestive heart failure [19]. Despite the differences in the study protocol and, inevitably, in the pharmacological treatment that the two cohorts of patients received before and after transplant, the results of the present and of the previous study are remarkably similar. In both studies, transplantation was associated with proportional decreases of pulmonary artery and of pulmonary capillary wedge pressure, meaning that the severity of pulmonary hypertension in such patients is mainly determined by the elevated venous pressure. In addition, both studies observed that mild alterations in the pulmonary circulation can persist early and late after cardiac transplantation (abnormalities in PCa in the present study and abnormalities in the pulmonary vascular closing pressure in the previous study). Unlike the previous study, however, the present study suggests that there is a particular hemodynamic profile which is associated with persistent hemodynamic abnormalities, i.e. a Cpc-PH profile. In the present cohort of patients, this profile identified patients in whom, possibly due to a long history of left heart disease, the mechanical effects of chronic venous congestion caused an additional component of pulmonary vascular disease. Interestingly, a small subgroup of patients with advanced heart failure had a pure pre-capillary hemodynamic profile; this is likely to be the result of an aggressive diuretic and vasodilator treatment which normalized the post-capillary component but was unable to modify the pre-capillary component of PH; this group seemed to behave as the Cpc-PH group, but due to the small number of patients it was not specifically analyzed.

### Limitations

This study included consecutive patients who were candidate for and underwent cardiac transplantation; patients with the most severe forms of PH, characterized by high levels of PVR and/or DPG, were not enrolled because currently these are hemodynamic contraindications to transplant surgery. Therefore, it was impossible to specifically explore the role of high DPG values, suggested to be a hemodynamic parameter specifically indicating heart failure patients with severe pulmonary vascular disease [3]. The prognostic role of high DPG is highly debated in the literature, but its diagnostic relevance has never been questioned [20–22]. This was a single center study based on a relatively low number of patients, which precluded the possibility of further analysis such as the evaluation of the impact of etiology, disease duration or of different treatments before transplantation. In addition, it would have been extremely interesting to explore the association of the lesser improvement in PCa in patients with Cpc-PH with morbidity and mortality after transplantation, even though the values of PCa observed after transplantation were well over the prognostic thresholds identified in heart failure patients [11–13]. However, the present study was not sized to explore such possibilities; future studies with larger sample size are necessary to test the hypothesis that a reduced PCa might predict cardiovascular events after heart transplantation. Finally, which type of pulmonary vascular disease is responsible for the persistent hemodynamic abnormalities remains unknown; unfortunately, few studies have analyzed pulmonary histopathology in Group 2 PH patients and no data have been published concerning its evolution after heart transplant [23].

### Conclusions

The analysis of pulmonary arterial compliance in patients with advanced congestive heart failure suggests that a hemodynamic profile characterized by Cpc-PH is associated with the persistence of a stiffer pulmonary circulation at 1 year after transplantation.

## Supporting information

S1 Dataset This is the excel file including individual data before and after tranplant. (XLSX)

## References

[1] Ponikowski P, Voors AA, Anker SD, Bueno H, Cleland JG, Coats AJ et al Authors/Task Force Members. 2016 ESC Guidelines for the diagnosis and treatment of acute and chronic heart failure: The Task Force for the diagnosis and treatment of acute and chronic heart failure of the European Society of Cardiology (ESC)Developed with the special contribution of the Heart Failure Association (HFA) of the ESC. Eur Heart J. 2016;37:2129–2200. 10.1093/eurheartj/ehw128 27206819 10.1093/eurheartj/ehw128

[2] Naeije R, Vachiery J, Yerly P, Vanderpool R. The trans]pulmonary pressure gradient for the diagnosis of pulmonary vascular disease. Eur Respir J. 2013;41:217–223. 10.1183/09031936.00074312 22936712 10.1183/09031936.00074312

[3] Gerges C, Gerges M, Lang MB, Zhang Y, Jakowitsch J, Probst P, et al Diastolic pulmonary vascular pressure gradient: A predictor of prognosis in “out-of-proportion” pulmonary hypertension. Chest. 2013;143:758–766. 10.1378/chest.12-1653 23580984 10.1378/chest.12-1653

[4] Bonderman D, Martischnig AM, Vonbank K, Nikfardjam M, Meyer B, Heinz G, et al Right ventricular load at exercise is a cause of persistent exercise limitation in patients with normal resting pulmonary vascular resistance after pulmonary endarterectomy. Chest. 2011;139:122–127. 10.1378/chest.10-0348 20671059 10.1378/chest.10-0348

[5] Ghio S, Morsolini M, Corsico A, Klersy C, Mattiucci G, Raineri C, et al Pulmonary arterial compliance and exercise capacity after pulmonary endarterectomy. Eur Respir J. 2014;43:1403–1409. 10.1183/09031936.00195313 24435007 10.1183/09031936.00195313

[6] Gan CT, Lankhaar JW, Westerhof N, Marcus JT, Becker A, Twisk JW, et al Noninvasively assessed pulmonary artery stiffness predicts mortality in pulmonary arterial hypertension. Chest. 2007;132:1906–1912. 10.1378/chest.07-1246 17989161 10.1378/chest.07-1246

[7] Sanz J, Kariisa M, Dellegrottaglie S, Prat-González S, Garcia MJ, Fuster V, et al Evaluation of pulmonary artery stiffness in pulmonary hypertension with cardiac magnetic resonance. JACC Cardiovasc Imaging. 2009;2:286–295. 10.1016/j.jcmg.2008.08.007 19356573 10.1016/j.jcmg.2008.08.007

[8] Mahapatra S, Nishimura RA, Sorajja P, Cha S, McGoon MD. Relationship of pulmonary arterial capacitance and mortality in idiopathic pulmonary arterial hypertension. J Am Coll Cardiol. 2006;47:799–803. 10.1016/j.jacc.2005.09.054 16487848 10.1016/j.jacc.2005.09.054

[9] Ghio S, D'Alto M, Badagliacca R, Vitulo P, Argiento P, Mulè M, et al Prognostic relevance of pulmonary arterial compliance after therapy initiation or escalation in patients with pulmonary arterial hypertension. Int J Cardiol. 2017 3 1;230:53–58. 10.1016/j.ijcard.2016.12.099 28038821 10.1016/j.ijcard.2016.12.099

[10] Lankhaar JW, Westerhof N, Faes TJ, Gan CT, Marques KM, Boonstra A, et al Pulmonary vascular resistance and compliance stay inversely related during treatment of pulmonary hypertension. Eur Heart J. 2008;29:1688–1695. 10.1093/eurheartj/ehn103 18349027 10.1093/eurheartj/ehn103

[11] Miller WL, Grill DE, Borlaug BA. Clinical features, hemodynamics, and outcomes of pulmonary hypertension due to chronic heart failure with reduced ejection fraction. JACC Heart Fail. 2013;1:290–299. 10.1016/j.jchf.2013.05.001 24621932 10.1016/j.jchf.2013.05.001

[12] Dupont M, Mullens W, Skouri HN, Abrahams Z, Wu Y, Taylor DO, et al Prognostic role of pulmonary arterial capacitance in advanced heart failure. Circ Heart Fail. 2012;5:778–785. 10.1161/CIRCHEARTFAILURE.112.968511 23087402 10.1161/CIRCHEARTFAILURE.112.968511 PMC3538355

[13] Pellegrini P, Rossi A, Pasotti M, Raineri C, Cicoira M, Bonapace S, et al Prognostic relevance of pulmonary arterial compliance in patients with chronic heart failure. Chest. 2014;145:1064–1070. 10.1378/chest.13-1510 24356904 10.1378/chest.13-1510

[14] Reuben SR. Compliance of the pulmonary arterial system in disease. Circ Res. 1971;29:40–50. 5561407 10.1161/01.res.29.1.40

[15] Lankhaar JW, Westerhof N, Faes TJ, Marques KM, Marcus JT, Postmus PE, et al Quantification of right ventricular afterload in patients with and without pulmonary hypertension. Am J Physiol Heart Circ Physiol. 2006; 291:H1731–H1737. 10.1152/ajpheart.00336.2006 16699074 10.1152/ajpheart.00336.2006

[16] Saouti N, Westerhof N, Postmus PE, Vonk-Noordegraaf A. The arterial load in pulmonary hypertension. Eur Respir Rev. 2010;19:197–203. 10.1183/09059180.00002210 20956192 10.1183/09059180.00002210 PMC9487275

[17] Tedford RJ, Hassoun PM, Mathai SC, Girgis RE, Russell SD, Thiemann DR, et al Pulmonary capillary wedge pressure augments right ventricular pulsatile loading. Circulation. 2012;125:289–297. 10.1161/CIRCULATIONAHA.111.051540 22131357 10.1161/CIRCULATIONAHA.111.051540 PMC3264431

[18] Masri SC, Tedford RJ, Colvin MM, Leary PJ, Cogswell R. Pulmonary Arterial Compliance Improves Rapidly After Left Ventricular Assist Device Implantation. ASAIO J. 2017; 63:139–143. 10.1097/MAT.0000000000000467 27831997 10.1097/MAT.0000000000000467

[19] Naeije R, Lipski A, Abramowicz M, Lejeune P, Mélot C, Antoine M, et al Nature of pulmonary hypertension in congestive heart failure. Effects of cardiac transplantation. Am J Respir Crit Care Med. 1994;149:881–887. 10.1164/ajrccm.149.4.8143050 8143050 10.1164/ajrccm.149.4.8143050

[20] Tedford RJ, Beaty CA, Mathai SC, Kolb TM, Damico R, Hassoun PM, et al Prognostic value of the pre-transplant diastolic pulmonary artery pressure-to-pulmonary capillary wedge pressure gradient in cardiac transplant recipients with pulmonary hypertension. J Heart Lung Transplant. 2014;33:289–297. 10.1016/j.healun.2013.11.008 24462554 10.1016/j.healun.2013.11.008 PMC3955214

[21] Tampakakis E, Leary PJ, Selby VN, De Marco T, Cappola TP, Felker GM, et al The Diastolic Pulmonary Gradient Does Not Predict Survival in Patients With Pulmonary Hypertension Due to Left Heart Disease. JACC Heart Fail. 2015;3:9–16. 10.1016/j.jchf.2014.07.010 25453535 10.1016/j.jchf.2014.07.010 PMC4289416

[22] Naeije R. Measurement to Predict Survival: The case of diastolic pulmonary gradient. J Am Coll Cardiol HF. 2015; 5:425. 10.1016/j.jchf.2014.12.014 25951765

[23] Delgado JF, Conde E, Sánchez V, López-Ríos F, Gómez-Sánchez MA, Escribano P, et al Pulmonary vascular remodeling in pulmonary hypertension due to chronic heart failure. Eur J Heart Fail. 2005; 7:1011–1016. 10.1016/j.ejheart.2004.10.021 16227139 10.1016/j.ejheart.2004.10.021

