# Correction: Persistent abnormalities in pulmonary arterial compliance after heart transplantation in patients with combined post-capillary and pre-capillary pulmonary hypertension

**DOI:** 10.1371/journal.pone.0208863

**Published:** 2018-12-04

**Authors:** Stefano Ghio, Gabriele Crimi, Silvia Pica, Pier Luigi Temporelli, Massimo Boffini, Mauro Rinaldi, Claudia Raineri, Laura Scelsi, Massimo Pistono, Rossana Totaro, Stefania Guida, Luigi Oltrona Visconti

## Notice of republication

An incorrect version of S1 Dataset was published in error. This article was republished on November 19, 2018 to correct for this error. Please download this article again to view the correct version.

## References

[1] Ghio S, Crimi G, Pica S, Temporelli PL, Boffini M, Rinaldi M, et al (2017) Persistent abnormalities in pulmonary arterial compliance after heart transplantation in patients with combined post-capillary and pre-capillary pulmonary hypertension. PLoS ONE 12(11): e0188383 10.1371/journal.pone.0188383 29176890 PMC5703525

